# Recent Advances in Titanium-Based Metal–Organic Frameworks: Structure, Property, and Application in Photocatalysis

**DOI:** 10.3390/molecules31050872

**Published:** 2026-03-05

**Authors:** Pengcheng Xing, Boxuan Yang, Lingshi Meng, Tianqi Jia, Shengjie Wang

**Affiliations:** College of Chemistry and Chemical Engineering, China University of Petroleum, Qingdao 266580, China; z24030181@s.upc.edu.cn (P.X.); boxuany2000@163.com (B.Y.); z24030168@s.upc.edu.cn (L.M.); 15553145064@163.com (T.J.)

**Keywords:** titanium-based metal–organic frameworks, synthesis strategies, structure–property relationship, photocatalysis

## Abstract

Metal–organic frameworks (MOFs) possess ordered pore structure, high surface area, tunable composition and tailorable functionality, and thus present promising prospect in many applications. Among them, titanium-based MOFs (Ti-MOFs) composed of organic ligands and titanium–oxygen clusters exhibit great potential in photocatalysis, owing to their diverse topological configurations, outstanding photocatalytic activity, low toxicity, and easy production. The latest developments in Ti-MOFs, including the synthetic strategies, structural features, methods for enhancing catalytic performance, and typical applications, were reviewed in this paper. The application in CO_2_ reduction, hydrogen evolution, organic pollutant removal, and photocatalytic sensing were emphasized. Moreover, we present a distinctive perspective on the relationship between the structure and their applications of Ti-MOFs, and provide new information in the design and construction of advanced Ti-MOFs for high-efficiency photocatalytic conversion.

## 1. Introduction

Metal–organic frameworks (MOFs) present unique structural properties that stem from the self-assembly of organic ligands with metal ions or clusters [1,2,3]. Compared to traditional porous materials, MOFs exhibit distinctive advantages, including high porosity, large specific surface areas, regular and tunable pore structures, and diverse topological configurations [4,5]. Till now, more than 90,000 kinds of MOFs have been constructed, containing almost all metals [6]. Among them, Ti-MOFs containing organic ligands and titanium–oxygen clusters have attracted special attention due to their rich structural topologies, outstanding photocatalytic activity, low toxicity, and relatively lower producing cost [7]. These properties allow them to be useful in many fields, including gas separation [8], gas storage [9], catalysis [10], and sensing [11]. Ti-MOFs exhibit significant advantages in photocatalysis due to their unique photoredox properties and visible-light responsiveness. This opens new possibilities for environmental remediation and renewable energy conversion (Table 1). Compared to semiconductor TiO_2_, a widely used commercial photocatalyst, Ti-MOFs possess distinct advantages [10]. For example, TiO_2_ has a relatively large bandgap, rendering it active only under ultraviolet irradiation. Meanwhile, its specific surface area, active sites, and pore structure are difficult to precisely control [12,13]. In contrast, Ti-MOFs allow flexible adjustment of bandgap and electro-structures to adapt to specific photocatalytic reactions. This process enhances light-harvesting efficiency, improves photoelectric conversion efficiency, and suppresses electron–hole recombination [14]. Consequently, they exhibit boosting catalyst activity and excellent stability, enabling them to be efficient and recyclable photocatalysts [15].

Despite the undeniable challenges in the synthesis process, Ti-MOFs have demonstrated great potential in photocatalytic hydrogen production [16], carbon dioxide reduction [17], and organic pollutant degradation [18]. Although previous reviews have summarized synthetic methods and applications, recent investigations have provided deeper insights into the relationship between structure and photocatalytic performance. Therefore, the latest strategies for synthesizing stable and advanced Ti-MOFs and significant advances in the photocatalytic applications were included in this review. The key factors that determine catalytic efficiency were focused on. This provides more valuable information for authors and gives new inspiration and guidance for the design and construction of high-performance Ti-MOFs.

**Table 1:** Composition, bandgap, and photocatalytic performances of different Ti-MOFs.

Ti-MOF	Ti-Oxo-Cluster	Bandgap (eV)	Application	Catalytic Performance	Ref.
MIL-125	Ti_8_O_8_(OH)_4_(CO_2_)_16_	3.60	Alcohol Oxidation	92%/>99%	[19,20]
MIL-125-NH_2_	Ti_8_O_8_(OH)_4_(CO_2_)_16_	2.60	CO_2_ Photo reduction, H_2_ Production	4.8 μmol·g^−1^·h^−1^	[21]
PCN-415	Ti_8_Zr_2_O_12_(COO)_16_	3.30	H_2_ Production	44 μmol·g^−1^·h^−1^	[22]
ACM-1	(Ti-O_2_)ₙ	2.30	Cyclohexanol Oxidation	52%/100%	[23,24]
ZSTU-1	(Ti_6_O_12_)ₙ	2.30	H_2_ Production	1060 μmol·g^−1^·h^−1^	[25]
DGIST-1	(Ti-O)ₙ	1.85	Alcohol Oxidation	93%/>99.5%	[26,27]
ZSTU-3	(Ti_6_O_12_)ₙ	2.20	H_2_ Production	1350 μmol·g^−1^·h^−1^	[25]
PCN-22	Ti_7_O_6_(COO)_12_	1.93	Alcohol Oxidation	28%/100%	[26,28]
MOF-901	Ti_6_O_6_(COO)_6_	2.65	Methyl Methacrylate Polymerization	87%	[29,30]
MOF-902	Ti_6_O_6_(COO)_6_	2.50	Benzyl Methacrylate Polymerization	68%	[31]
FIR-125	Ti_8_O_8_(OH)_4_(COO)_16_	2.95	Propane Selective Adsorption	39.5 cm^3^·g^−1^	[32,33]
MIL-101-Ti	Ti_3_O(COO)_6_	-	O_2_ Irreversible Chemisorption	1.1 mmol·g^−1^	[34]

## 2. Structural Characteristics of Ti-MOF

### 2.1. Ordered Network Architecture

**Figure 1:**
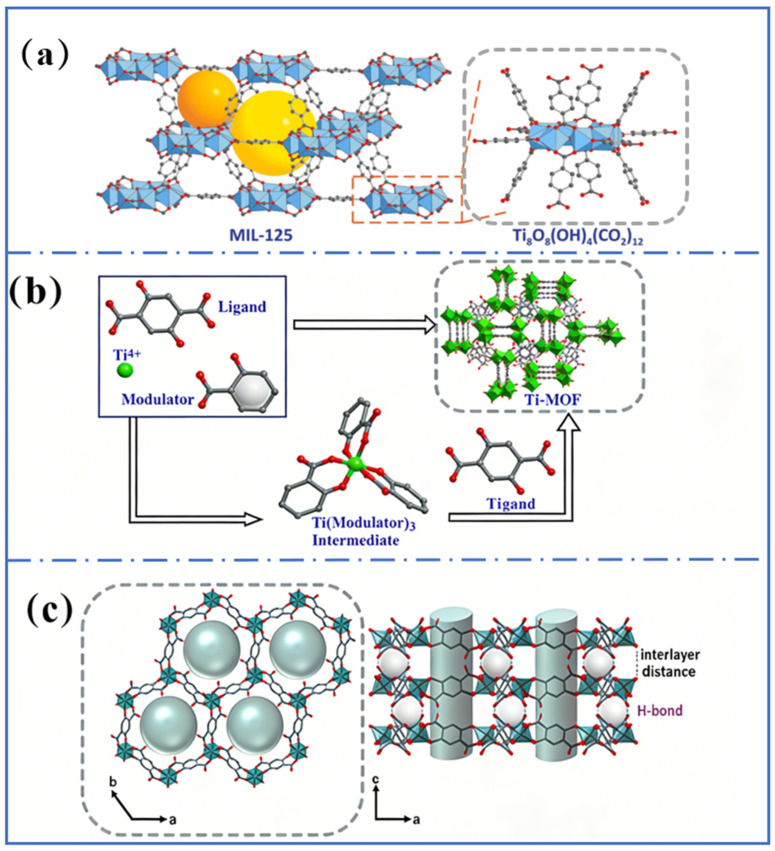
(**a**) Crystal structure of MIL-125, shown in a 3D view with octahedral and tetrahedral pores represented by yellow and orange spheres, respectively. The 12-c of the octahedral ring in Ti_8_O_8_(OH)_4_(CO_2_)_12_ is also depicted on the right. Reproduced with permission [35]. (**b**) Single-crystal structure of FIR-119. Reproduced with permission [33]. (**c**) Image of the crystal structure of NTU-9, along the pore (view along c-axis) and visualization of the layer structure (view along b-axis), with the pores and pore content, i.e., positionally disordered solvent molecules. Reproduced with permission [40].

Ti-MOFs possess ordered network architectures and highly regulated porous characteristics, which originate from the self-assembly of titanium metal centers with organic ligands. For instance, MIL-125 (Ti) was reported to have a three-dimensional pore structure where titanium–oxygen clusters were connected with terephthalic acid ligands to form octahedral coordination configurations (Figure 1a) [35]. This ordered network endows MIL-125 (Ti) with a large specific surface area (3344 m^2^/g) and pore volume (0.56 cm^3^/g), providing favorable conditions for the adsorption and diffusion of guest molecules in air [36]. FIR-119, constructed by titanium ions and 2,5-dihydroxyterephthalic acid ligands, exhibited a chiral self-reticulating square lattice topology (Figure 1b), providing suitable channels for gas molecule separation [33]. The ordered network structure of Ti-MOFs can be regulated and engineered by the selection of metal nodes, organic ligands, and synthesis conditions. Change in ligand length [37] or functional groups [38] results in tunable pore size and channel geometry, thereby influencing their adsorption and catalytic performances [39].

### 2.2. Abundant Metal Nodes

**Figure 2:**
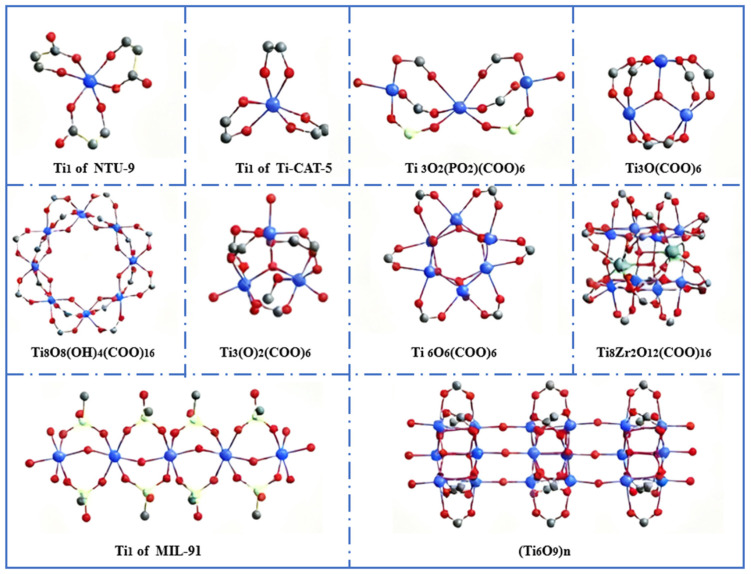
Different Ti-O nodes involved in Ti-MOFs. Color code: Ti, blue; O, red; C, gray; P, white; Zr, green. Reproduced with permission [43].

Various metal nodes of Ti-MOFs exhibit unique characteristics. Titanium ions exhibit high reactivity and oxygen affinity. This endows them with chemical reactivity and facilitates the synthesis of Ti-MOFs to some extent [41]. Intricate frameworks can be constructed by various metal coordination structures (Figure 2), which is essential for their diverse functionalities and applications. Structural diversification of Ti-MOFs can also be achieved through changing organic ligands or incorporating hetero-metallic ions. For instance, NTU-9 exhibited a hexagonal prism morphology where each Ti^4+^ ion was connected to three organic ligands to form TiO_6_ clusters [42]. NTU-9 comprised two-dimensional layers with a honeycomb (hcb) network, where six Ti-O clusters form hexagonal layers first (Figure 1c). These layers stacked upon each other via hydrogen bonding interactions to generate a pseudo-three-dimensional structure. The interlayer spacing between adjacent layers was approximately 6 Å, with one-dimensional channels about 11 Å. NTU-9 exhibited an absorption spectrum spanning from 400 nm to 750 nm, with an absorption peak centered around 520 nm, consistent with its low bandgap (1.74 eV). Compared to indirect bandgap semiconductors like TiO_2_, NTU-9 showed higher photon conversion efficiency.

Taking the classical MIL-125 as an example [44], Ti_8_O_8_(OH)_4_-(O_2_C-C_6_H_4_-CO_2_)_6_ presented an orthorhombic face-centred cubic (fcu) topology constructed from Ti_8_O_8_(OH)_4_ clusters and 1,4-benzenedicarboxylate linkers. MIL-125 comprised ring-shaped octahedra connected via corner- or edge-sharing, formed by eight TiO_6_ octahedral titanium units bonded to oxygen atoms (Figure 1a). These octahedra were linked to 12 twisted cubic clusters via benzene 1,4-dicarboxylate linkers to form a porous 3D quasi-cubic tetrahedral structure. Highly stable chemical bonds are formed between the titanium–oxygen clusters and the phenyl dicarboxylate linkers, which provide a stable framework for the metallic nodes. This demonstrates the effect of metal nodes on material functionality.

For another classic Ti-MOF, PCN-415, the secondary building unit consisted of a central Ti_8_(m-O)_4_ cube in which Ti(IV) atoms are located at the vertices [45]. Two Zr(IV) atoms were positioned on opposite faces of the Ti_8_ cube and connected to the Ti ions via eight μ_3_-O^2−^ ions to form a [Ti_8_Zr_2_(μ-O)_4_(μ_3_-O)_8_]^16+^ core. These metal nodes exhibit high catalytic activity. They serve as a catalyst in certain photocatalytic reactions to promote the separation and transfer of photo-generated charges, and thus enhance the photocatalytic efficiency. These properties stem from the unique structure and chemical characteristics of the metal nodes, which endow Ti-MOFs with promising potential in gas adsorption [46] and photocatalysis [47].

## 3. Synthesis Methods of Ti-MOFs

### 3.1. Solvothermal Synthesis

**Figure 3:**
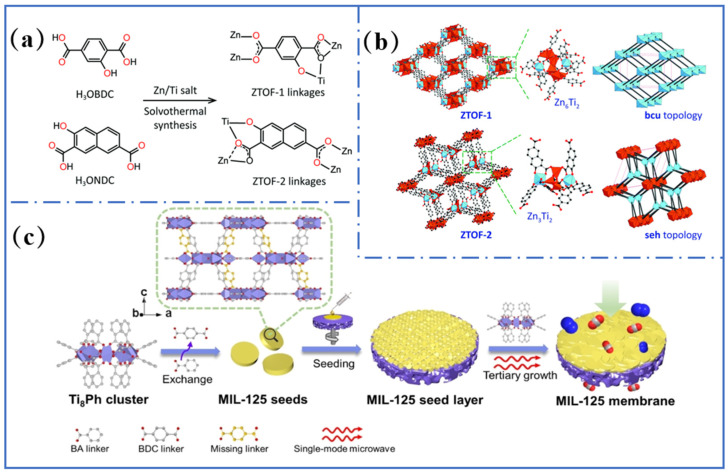
(**a**) Zn/Ti-oxo clusters in ZTOF-1 and ZTOF-2. Reproduced with permission [35]. (**b**) Representative structures in ZTOF-1 and ZTOF-2. Reproduced with permission [35]. (**c**) Schematic illustration of the preparation of defect-containing MIL-125 films via a combination of single-mode microwave heating and three-stage growth. Reproduced with permission [42].

Most Ti-MOFs are prepared by solvothermal synthesis. Titanium precursors, organic ligands, catalysts, and liquid organic solvents are placed in a sealed system. Various MOF materials can be obtained by rationally optimizing the synthesis parameters, including the titanium metal source [9], solvent [48], temperature [49], and reaction time [50]. Mono-metallic Ti-MOFs such as MIL-125 and NTU-9 were prepared using titanium isopropoxide as the precursor [20,40]. The Ti_8_O_8_ ring-like cluster in MIL-125 provides a crucial matrix for subsequent functionalization [51]. Auxiliary metals such as Zn and Co could be introduced in bimetallic MOF synthesis. ZTOF-1 and ZTOF-2 series materials were prepared by regulating crystallization kinetics through stepwise temperature ramping (Figure 3a,b) [35,52]. The advantage of the solvothermal method includes simple operation, abundant ligands, and the ability to synthesize diverse structures. Its disadvantage lies in the high reactivity of titanium salts, which often leads to excessively rapid reaction rates. This often produces microcrystals or powders, reducing the likelihood of well-defined crystals [48,49].

### 3.2. Microwave-Assisted Synthesis

Microwave-assisted synthesis utilizes high-frequency electromagnetic waves to achieve volumetric heating of reaction systems, overcoming the rate limitations of conventional conduction heating and emerging as a preferred technology for efficient synthesis. The advantage lies in its high heating uniformity, which shortens the time for crystallization and optimizes the morphology, making it particularly well-suited for the requirements of large-scale production [53]. For example, a highly continuous and dense MIL-125 membrane was fabricated under microwave-heated reaction conditions. This approach increased framework coordination vacancy density to enhance CO_2_/N_2_, H_2_/N_2_, and H_2_/CH_4_ separation efficiency and promoted preferential c-axis epitaxial growth of the MIL-125 membrane (Figure 3c) [42]. Similarly, flake-like NH_2_-MIL-125 was obtained by a microwave-solvent synergistic regulation strategy using ethanol as the solvent [54]. This method reduced the crystal thickness from micrometers to 200 nm, and thus exposed more surface-active Ti sites. The microwave heating method accelerates crystallization and enables precise tuning of MOF morphology and dimensions [55].

### 3.3. Supercritical Fluid Synthesis

**Figure 4:**
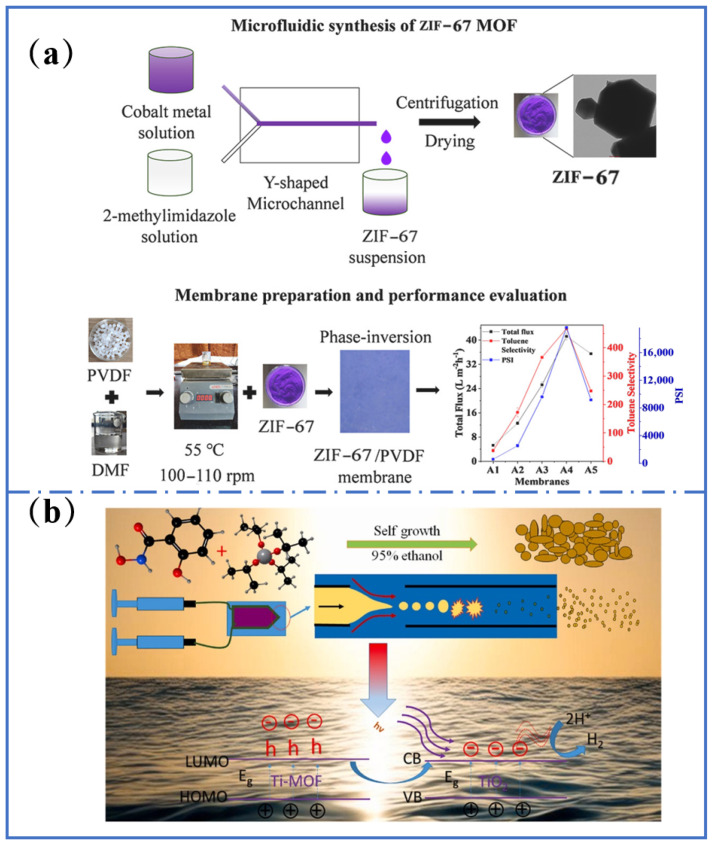
(**a**) Schematic of supercritical carbon dioxide fluid processing of ZIF-67 membranes. Reproduced with permission [65]. (**b**) Schematic presentation of the synthesis process of the novel MOF. Reproduced with permission [58].

Supercritical fluid synthesis (scCO_2_) technology has demonstrated significant advantages in Ti-MOF synthesis, particularly in controlling the morphology, purity, and scalability for large-scale synthesis. This technique uses CO_2_ under supercritical conditions as the solvent and reaction medium, which not only minimizes byproduct generation but also reduces environmental pollution and material synthesis costs [55]. Under the scCO_2_ environment, the Ti-MOF synthesis process can be precisely regulated by controlling parameters such as reactant ratios, reaction time, temperature, and pressure, in which all of them directly influence the structure and property of the final product [56]. Taking the novel MOF synthesis as an example, the ratio of ligand-to-metal ions was found to be crucial to the product purity. Ti-MOFs with high specific surface area and specific morphology were obtained under optimized conditions, which were crucial for the applications of hydrogen production (Figure 4a) [57,58]. Similarly, the morphology and properties of ZIF-67 could be controlled by regulating the reaction conditions (Figure 4b) [59].

Another feature of supercritical synthesis technology is its assurance of material homogeneity and reproducibility. Factors such as solvent evaporation and mixing inhomogeneity often lead to changes in the reaction environment in traditional solution synthesis [60]. However, the supercritical state of CO_2_ provides an exceptionally uniform solvent environment, ensuring uniform synthesis for each molecule—a critical factor for producing high-quality materials. The supercritical fluid synthesis method offers an environmentally friendly, highly controllable, and reproducible approach for the synthesis of MOFs. Future research should further explore this method in synthesizing different Ti-MOFs and investigate how to optimize the material properties.

### 3.4. Electrochemical Synthesis

Electrochemical synthesis focuses on enhancing the performance of Ti-MOFs by in situ loading of the active components onto the material surface. This method achieves functional upgrades while preserving the bulk structure [61]. Its core logic involves utilizing electron transfer reactions at the electrode surface to anchor functional units onto the surface of Ti-MOF, thereby constructing a carrier-active component synergistic system [62]. Using a titanium plate as the anode and a platinum plate as the cathode, constant-voltage electrolysis was performed in a DMF/ethanol mixed electrolyte, in which Ti^4+^ ions were generated by dissolving the anodic titanium sheet. Ti^4+^ coordinated with H_2_BDC ligands in the electrolyte and formed a dense MIL-125 (Ti) film on the electrode surface. The film thickness can be precisely controlled by the electrolysis time. The film exhibits preferential c-axis orientation due to its directional growth along the c-axis, significantly surpassing the generation of powder-pressed films [63]. An electrochemical in situ doping–oxidation two-step method was employed: First, Ti_3_C_2_Tx was used as the precursor, followed by cyclic voltammetry to reduce and dope Cu^2+^ into the MIL-125 framework. Subsequently, anodic oxidation converted Cu to Cu^2+^, yielding Cu-Ti-MIL-125 complexes [64]. A heterojunction of Cu^2+^ and Ti^4+^ was incorporated in this material, which extends the photo-generated electron–hole pair lifetime and increases the photocatalytic hydrogen evolution activity.

### 3.5. Mechanical Grinding Method

Traditional synthesis of MOFs often relies on high temperatures, high pressures, and large amounts of organic solvents, leading to huge energy consumption, significant pollution, high costs, and large quantities of byproducts [66]. In contrast, the mechanical grinding method involves grinding solid reagents at room temperature under solvent-free or minimal solvent conditions. The reactions are driven by physical mechanical forces, reducing carbon emissions and environmental impact and enabling room-temperature operation. This method achieves high reaction efficiency and enables Ti-MOF synthesis within a short timeframe [67]. MIL-125(Ti) or NH_2_-MIL-125(Ti) powders were directly obtained from titanium isopropoxide and terephthalic acid (or 2-aminoterephthalic acid) by mechanically grinding at room temperature [68]. X-ray diffraction confirms that pure-phase orthorhombic face-centred cubic topologies have been obtained. Similarly, Ti-MIL-101 with orthorhombic face-centred cubic structure can also be prepared using TiCl_3_ as the titanium source and terephthalic acid as the ligand by utilizing screw shear and extrusion pressure [69]. They show comparable specific surface area and pore volume to that of traditional solvothermal methods. More interestingly, continuous production is achieved using a twin-screw extruder, yielding over 50 g per hour. This approach provides a new pathway for large-scale, environmentally friendly preparation of Ti-MOFs.

### 3.6. Microfluidic Synthesis

Microfluidic synthesis technology leverages micron-scale channels to construct confined reaction environments. Continuous and highly uniform preparation of Ti-MOF can be achieved by precise control of reactant mixing rates, temperature, and residence time [70]. Bottlenecks in traditional batch synthesis, such as product inhomogeneity caused by concentration gradients and prolonged reaction cycles, can be overcome in this method. For example, Mabaleha et al. [71] developed a dual-droplet nested microfluidic system. MIL-125 was continuously synthesized within a 120 °C microreactor after a 30 min residence time. The particle size deviation is limited within 5% and the crystal size can be tuned from 200 nm to 1.2 μm by adjusting the droplet flow rate ratio. With continuous operation for 8 h, the product purity is maintained above 98%, providing a viable solution for industrial-scale continuous production of Ti-MOFs. Moreover, this method enhances the predictability of reaction kinetics while significantly reducing reagent consumption and waste emissions.

Despite the significant advantages of microfluidic synthesis, challenges including microchannel clogging, stringent sealing requirements, and flux matching issues during equipment scaling still exist. The future development of chemically resistant ceramic chips and intelligent online monitoring systems is expected to advance their widespread application in Ti-MOF production at an industrial scale.

## 4. Molecular Design of Ti-MOF for Enhanced Photocatalytic Performance

### 4.1. Improvement in Light Responsiveness

#### 4.1.1. Ligand Functionalization

**Figure 5:**
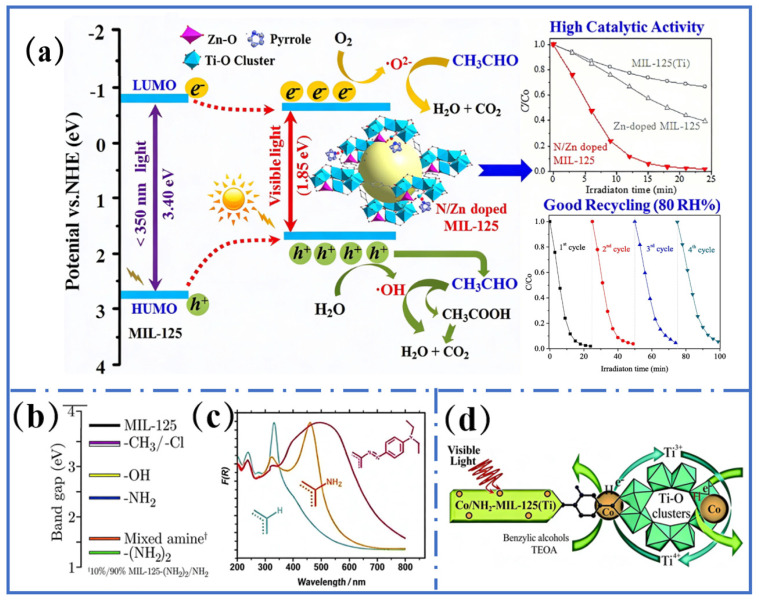
(**a**) Pyrrolic N/Zn co-doped MIL-125 (Ti) (with Ti/Zn-N/O clusters) enhances its photocatalytic activity (10× faster) and cycling stability for acetaldehyde degradation under high humidity. Reproduced with permission [79]. (**b**) Bandgaps of MIL-125 (black) and its analogs containing different functional linkers. Reproduced with permission [72]. (**c**) UV diffuse reflectance spectra of Ti-MOF materials: MIL-125 (Ti) (gray), NH_2_-MIL-125 (Ti) (orange), and MR-MIL-125 (Ti) (red). Reproduced with permission. [75]. (**d**) Proposed mechanism for photocatalytic CO_2_ reduction over Co/NH_2_-MIL-125 (Ti) upon visible-light irradiation. Reproduced with permission [21].

The light absorption capacity of Ti-MOFs can be improved by ligand functionalization. Introduction of electron-donating groups such as -NH_2_ and -OH can regulate the energy level of ligands [72,73]. For instance, the bandgap of MIL-125-NH_2_ was reduced to 2.6 eV by replacing terephthalic acid with 2-aminoterephthalic acid. The light absorption edge redshifted from 350 nm to 550 nm (Figure 5b) [72]. The bandgap of Ti-MOFs (MIL-125-(NH_2_)_2_) was further reduced to 1.3 eV by introducing a double-amino ligand. This allows the material to absorb near-infrared light and enhance the utilization of long-wavelength illumination, resulting in a 40% enhancement in photocatalytic activity (Figure 5c) [73,74,75]. In addition, diazotization modification [76] and post modification [77] also contribute to expanding the light response spectrum of Ti-MOFs. The modified Ti-MOFs exhibit a delocalization effect due to the π electrons of the -OH group interacting with the π-conjugated system of the ligands. The absorption intensity in the 400–460 nm region increased threefold compared to that of the pristine material [78].

#### 4.1.2. Ion Doping

The light absorption property of Ti-MOFs can also be enhanced by ion doping. Part of the Ti^4+^ ions was replaced by Zn^2+^ ions in a N/Zn co-doped MIL-125 material, which reduced the binding energy of Ti and increased the Ti^3+^/Ti^4+^ ratio. Simultaneously, the introduction of N created additional oxygen vacancies. This synergistic effect narrowed the bandgap of the material and resulted in a 98% degradation rate of acetaldehyde under visible light (Figure 5a) [79]. Loading noble metal nanoparticles like Pt or Ag on MOFs can improve the visible-light absorption capacity and light-capturing efficiency through the Schottky barrier or surface plasmon resonance (SPR) effect of metal nanoparticles [80]. For instance, Ag/MIL-125 composites achieved over 99% degradation of Rhodamine B within 40 min of visible-light irradiation, significantly surpassing unloaded samples (~8%), demonstrating the pronounced advantage of structural synergistic modification [81]. Electron transport pathways are established by the coordination bond coupling between metal nodes and ligands. Charge separation efficiency and visible-light absorption ability of the composites can be further enhanced by constructing heterojunctions. These characteristics endow Ti-MOFs with great potential in photocatalytic applications [81]. Co^2+^ acted as an electron mediator to promote charge transfer from ligands to metal nodes in Co-doped MIL-125-NH_2_, which enhanced visible-light-capturing ability and achieved a high reduction rate of CO_2_ to HCOOH (Figure 5d) [21]. To optimize its electronic structure through metal doping, Fe(NO_3_)_3_·9H_2_O was incorporated during Ti-MOF-74 synthesis. This allowed Fe^3+^ to partially replace Ti^4+^ within the Ti cluster framework and yielded Fe-doped Ti-MOF-74 (Fe-Ti-MOF-74). The bandgap of the material was reduced from 3.3 eV to 2.5 eV and at the same time, the light absorption edge redshifted from 376 nm to 496 nm by the synergistic effect of Fe^3+^ and Ti^4+^ [82,83]. Non-metallic doping proves equally effective. For instance, N-doped MIL-125-derived TiO_2_ (MM-500) forms a rutile–perovskite mixed phase after annealing at 500 °C. N atoms replace lattice oxygen to form impurity levels, resulting in the bandgap being reduced to 2.77 eV [84].

#### 4.1.3. Heterostructure Construction

Matching energy levels between different semiconductors may expand the light absorption range of materials. NH_2_-UiO-66 possessed a highly stable cubic structure with an intrinsic bandgap of 3.1 eV and exhibits poor visible-light absorption behavior [85]. Au nanoseeds were adsorbed onto the surface of NH_2_-UiO-66 to enhance light absorption via surface plasmon resonance (SPR) effects. Subsequently, HAuCl_4_ was reduced using sodium citrate to prepare Au/NH_2_-UiO-66 heterostructures. Au nanoparticles exhibit a strong SPR absorption peak at 520 nm, and the resulting intensive local electric field enhances light absorption intensity [86]. CdS/MIL-125 heterojunctions were formed by combining MIL-125 with narrow-bandgap semiconductors such as CdS (~2.4 eV). The light absorption range was extended to 600 nm via interfacial charge transfer, and a higher conversion rate for the oxidation of benzyl alcohol to benzaldehyde was achieved [87]. Similarly, other semiconductors like g-C_3_N_4_ and noble metals such as Ag have been incorporated with Ti-MOFs to improve their light-capturing capacity [88,89]. These approaches provide crucial information for the design of highly efficient photocatalytic materials.

### 4.2. Improvement in Charge Separation and Transfer

#### 4.2.1. Regulation of Crystal Structure

Regulation of the crystal structure of materials provides a possible methodology for enhancing charge transfer efficiency. In the orthorhombic face-centred cubic topology of MIL-125, the interactions between Ti_8_O_8_(OH)_4_ clusters and terephthalic acid formed a 3D porous framework that not only increased the specific surface area but also constructs continuous electron transport pathways. This reduced the transport distance of photo-generated carriers within the bulk phase [90]. Two-dimensional MIL-125 nanosheets were synthesized by controlling the reaction conditions, in which the charge transport distance was further shortened and thus the electron–hole recombination rates were reduced. This results in a twofold increase in photocatalytic hydrogen evolution rates compared to bulk materials [91]. Mesoporous modifications such as the mesoporous core–shell heterojunction of MIL-125-NH_2_@Bi_2_MoO_6_ accelerated the diffusion of reactant and product through mesoporous channels and at the same time, enabled efficient charge transfer across the core–shell interface. This configuration endowed the material with a 93.28% degradation rate for dichlorophenol [92].

#### 4.2.2. Construction of Heterostructure

**Figure 6:**
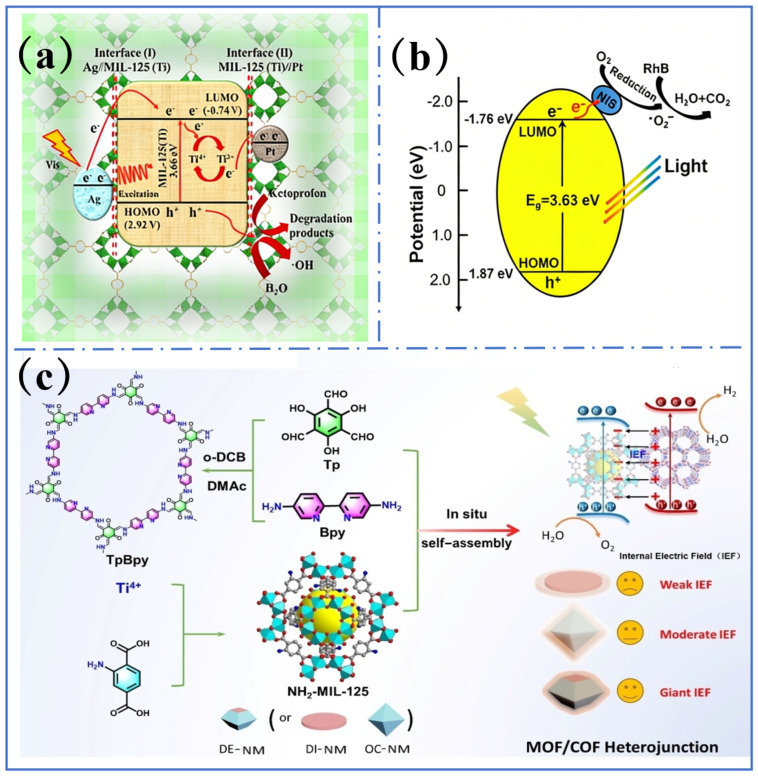
(**a**) Mechanism illustration for the photocatalytic process by Pt/MIL-125(Ti)/Ag. Reproduced with permission [93]. (**b**) Schematic illustration of the photocatalytic process of NS/MIL-x. Reproduced with permission [94]. (**c**) Synthesis of NH_2_-MIL-125(Ti)/TpBpy-COF. Reproduced with permission [97].

Constructing heterojunctions with a matched bandgap can promote charge separation. In Z-type heterojunctions such as Ag/NH_2_-MIL-125/CdS, the misalignment of the band between CdS (CB = −0.6 eV) and MIL-125 (CB = −0.2 eV) enabled photoexcited electrons to transfer from the CB of CdS to the CB of MIL-125, while holes moved to the VB of CdS. This effectively suppressed the recombination of photo-generated electrons and holes (Figure 6a) [93]. In a type-II heterojunction such as NiS/MIL-125, the VB potential of NiS (1.8 eV) was higher than that of MIL-125 (3.1 eV). Photo-generated holes transferred from the VB of MIL-125 to the VB of NiS, while electrons moved to the CB of MIL-125. Electrochemical impedance spectroscopy showed a 40% reduction in charge transfer resistance compared to the pristine MIL-125 (Figure 6b) [94]. Similarly, inhibited recombination of the photo-generated electron–hole pairs was observed in S-type heterojunctions (Figure 6c). This leads to a significant increase in photocatalytic efficiency in the fields of organic degradation [95,96,97], hydrogen evolution [16] and so on.

#### 4.2.3. Loading of Cocatalyst

The loading of cocatalyst can accelerate the charge transfer by capturing photo-generated charge carriers. For instance, loading of cocatalysts such as Pt and CoPi on the surface of MIL-125 was used to construct MIL-125/CoPi-Pt, in which Pt was used as a hydrogen evolution site to capture electrons while CoPi acted as an oxygen evolution site to capture holes [98]. This synergy facilitated the rapid separation of photo-generated electron–hole pairs. The overall water splitting rates for hydrogen and oxygen production reached 42.33 μL·h^−1^ and 21.33 μL·h^−1^, respectively, representing a 25-fold increase over pristine MIL-125 [44]. NiPd alloy was loaded on the surface of g-C_3_N_4_/MIL-125 to construct a complex photocatalyst. NiPd acts as an electron trap to promote charge accumulation, helping to achieve a hydrogen evolution rate of 8.7 mmol·g^−1^·h^−1^ [99]. Furthermore, encapsulating CuO quantum dots within MIL-125 and coupling them with g-C_3_N_4_ enabled rapid transfer of photoexcited electrons from MIL-125 or g-C_3_N_4_ to CuO. This ternary composite system achieves a CO_2_ reduction rate of 997.2 μmol·g^−1^, significantly outperforming binary systems [88]. These findings offer novel insights for enhancing charge separation and transfer efficiency and final photocatalytic performance.

### 4.3. Improvement in Stability

#### 4.3.1. Reinforcement of Metal–Ligand Coordination

Enhancing metal–ligand coordination is an effective method to improve the stability of MOF materials. For example, MIL-125 with uniform crystal particle size and reduced defects was obtained by a microwave-assisted synthesis method. This material remains intact across the pH range of 2–10, whereas MIL-125 synthesized via conventional solvothermal methods collapsed at pH > 8 [100]. Furthermore, more stable metal–ligand bonds were formed in Ti-MOF materials by dual-metal coordination. For example, Zr^4+^ ions have similar ionic radii and charges to Ti^4+^ in Ti-Zr-MIL-125-NH_2_. The incorporation of Zr^4+^ ions allowed them to partially replace Ti^4+^ ions in the framework and form stronger metal coordination bonds. Morphology and catalytic performance remained essentially unchanged after five cycles of degradation of paracetamol under simulated sunlight irradiation [101]. Ti-Zr mixed metal cluster MOFs were constructed by introducing 20% of molar ratio of Ti^4+^ into the Zr_6_O_4_(OH)_4_ cluster of PCN-22. The high electronegativity of Ti^4+^ enhanced the coordination with porphyrin ligands, shortened the lengths of metal–ligand bonds by 0.02 Å and increased the bond energy by 15%. Therefore, the hybrid clusters exhibited more uniform electron density distribution, which reduces proton attack sites under acidic/alkaline conditions, enhancing structural stability [28].

#### 4.3.2. Surface Modification

Surface modification mainly reduces the external erosion by constructing protective layers. For example, the unique framework of DGIST-1 is prone to destabilization due to the attack of water molecules in aqueous-phase catalysis [102]. To address this problem, researchers developed a one-step surface polymerization method to coat the particle with a hydrophobic polymer protective layer. The modified DGIST-1 exhibited virtually unchanged specific surface area in corrosive media (strong acids or strong bases) and high-temperature environments. Experiments demonstrated that this core–shell structure had no significant changes in crystal structure after 24 h of immersion in boiling water, whereas the uncoated framework had partially degraded [103]. Furthermore, coating MIL-125 with silica (SiO_2_) to form a core–shell structure prevented the penetration of water molecules into the MOF. The BET-specific surface area retention was about 90% after 24 h immersion in boiling water, while only 45% was obtained for the uncoated MIL-125 [104].

## 5. Photocatalytic Applications

### 5.1. Photocatalytic Hydrogen Production

**Figure 7:**
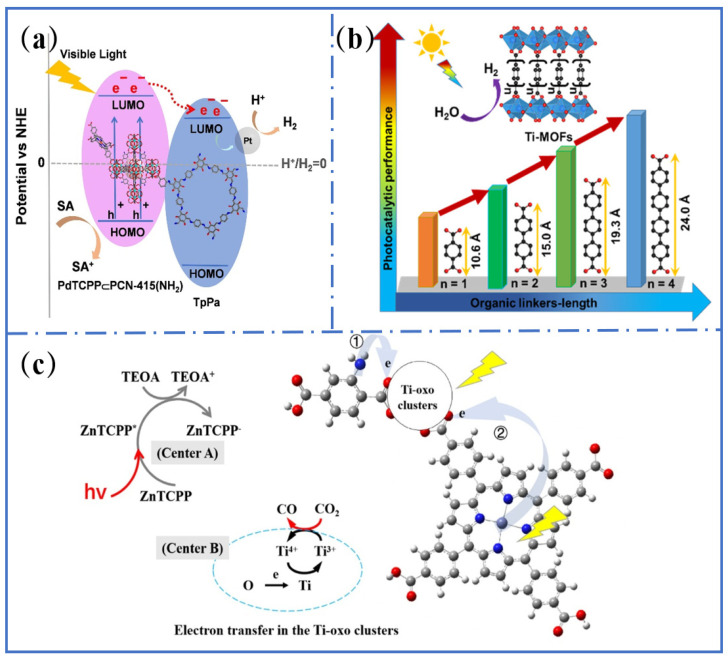
(**a**) The photocatalytic H_2_ evolution mechanism of composite 2. Reproduced with permission [112]. (**b**) Catalytic H_2_ evolution rates of nano-titanium metal–organic frameworks (MOFs) with aromatic carboxylic acid ligands of varying lengths. Reproduced with permission [26]. (**c**) Reaction scheme of D-TiMOF in photocatalytic CO_2_ reduction. (path 1: photoelectrons from excited NH_2_BDC; path 2: photoelectrons from excited ZnTCPP). Reproduced with permission [114].

Hydrogen energy is a renewable, zero-carbon-emission fuel, and environmental friendly energy source [105]. Among numerous preparation methods of hydrogen, photocatalytic hydrogen production has attracted great attention because solar energy is an inexhaustible and clean energy [106]. Therefore, the photocatalyst, as a key component in the photocatalytic system, has attracted more and more interest [107]. Ti-MOFs with highly ordered porous structures provide abundant adsorption sites and transport pathways for reactants and charges [108]. During the process of photocatalytic hydrogen evolution, water molecules and sacrificial electron donors (e.g., triethanolamine) rapidly diffuse through the pore to the surface of the catalyst and contact with the active sites [109]. Simultaneously, the pore structure also provides escape channels for the generated hydrogen, preventing excessive accumulation on the surface of the catalyst and thereby enhancing hydrogen evolution efficiency [110]. Furthermore, the pore architecture of Ti-MOFs can be optimized by adjusting pore size and channel geometry, thereby improving reactant adsorption and transport properties [111]. For instance, a series of multi-component Ti-MOF/COF hybrid materials (PDT CPP PCN-415 (NH_2_)/TPPA) was developed via a covalent integration strategy. These materials exhibited outstanding efficiency in photocatalytic hydrogen production. The results indicate that the synergistic interaction between the pore structure of Ti-MOF and the framework of COF optimizes the adsorption and transport of reactants, thereby enhancing their photocatalytic activity (Figure 7a) [112].

Titanium–oxygen clusters in Ti-MOFs serve as active sites for photocatalytic hydrogen evolution. These clusters exhibit excellent photocatalytic activity and can effectively catalyze the decomposition of water molecules to produce hydrogen. In Pt-deposited Ti-MOF-NH_2_ materials, the Pt nanoparticles synergistically interacted with the titanium centers to enhance the photocatalytic activity. As a cocatalyst, Pt nanoparticles promoted the separation and transfer of photo-generated carriers and thus reduced the electron–hole pair recombination. This enhanced the utilization of photo-generated carriers and boosted hydrogen evolution efficiency [113]. The ligand structure of Ti-MOFs also influences their photocatalytic hydrogen evolution performance. A series of structurally similar Ti-MOFs was synthesized with varying lengths. Their absorption band in the visible region gradually broadened and the bandgap became narrower when the ligands became longer (Figure 7b) [37].

### 5.2. Photocatalytic Reduction in CO_2_

Overuse of fossil fuels increases the levels of greenhouse gas CO_2_ and results in global warming, which poses a threat to the ecosystems [115]. Promising technologies of CO_2_ reduction include carbon capture and storage, electrochemical conversion, photoelectrochemical processes, and photocatalytic reduction [116,117,118,119,120]. CO_2_ and H_2_O can be converted to CH_4_, CO and other value-added compounds under sunlight irradiation [121]. Recently, Ti-MOFs have gained great attention in CO_2_ reduction due to their large surface area, tunable composition and structure, and tailorable functionality [122].

The titanium oxide clusters within Ti-MOFs serve as the primary active sites for photocatalytic CO_2_ reduction. Dual active sites were formed in D-TiMOF by the integration of zinc (II) tetrakis(4-carboxyphenyl)porphyrin [Zn-TCPP] and titanium–oxygen clusters (Figure 7c). The synergistic interaction between Zn-TCPP and the titanium–oxygen cluster facilitates electron transfer from Zn-TCPP to the titanium–oxygen cluster, significantly enhancing the photocatalytic reduction efficiency of CO_2_. The reduction rate of CO_2_ was about 59.55 µmol g^−1^ h^−1^, which is 11 times higher than that of Ti-MOF alone. This improvement in photocatalytic activity was attributed to D-Ti-MOF’s distinct band structure, enhanced CO_2_ absorption capacity, and synergy of the dual-metal active sites [114].

Doping with noble metal nanoparticles (e.g., Pt, Au) significantly enhances the photocatalytic performance of Ti-MOFs. These noble metal nanoparticles serve as redox reaction sites, reduce overpotentials of the reaction, and act as electron traps within the Ti-MOF pores [123]. Li et al. [124] reported that the synthesis of NH_2_-MIL-125 embedded with Pt and Au nanoparticles.Pt/NH_2_-MIL-125 exhibited higher photocatalytic activity, in which the yield of formic acid increased 21% compared to the pristine NH_2_-MIL-125. Such enhancement stems from the synergistic interactions between Pt nanoparticles and Ti-MOF, facilitating electron transfer and Ti^3+^ generation, thereby boosting photocatalytic efficiency in CO_2_ reduction.

### 5.3. Photocatalytic Degradation of Pollutants

More and more pollutants are discharged into the environment with industrialization and population growth. For the biodegradable pollutants, biological methods represent the most economical approach [125]. However, biological methods prove inadequate for certain refractory pollutants characterized by high molecular weight, poor degradability, and high toxicity. Adsorption and photocatalytic degradation technologies, characterized by high efficiency and broad applicability, serve as suitable alternatives or pre-treatment steps for biological methods [126,127]. Consequently, they hold promising prospects for treating organic pollutants, particularly for the recalcitrant ones. Notably, Ti-MOFs possess a broad light response range and an easily tunable structure and functionality, endowing them with distinct advantages in solar-powered photocatalytic pollution treatment.

**Figure 8:**
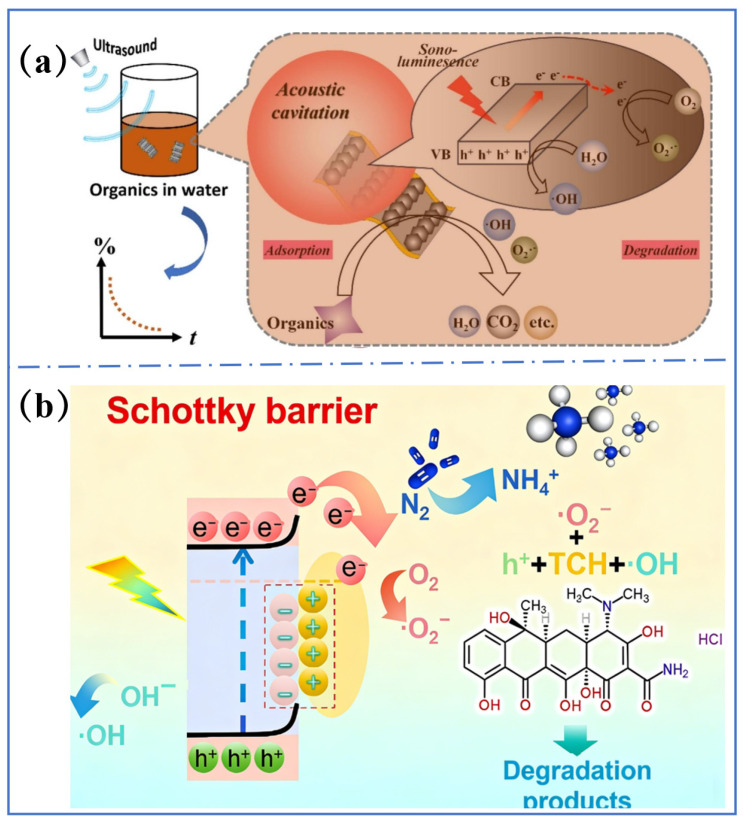
(**a**) Probable mechanism of MX@MIL-125(Ti/Zr)-mediated sonocatalytic removal for the Rhodamine B [129]. (**b**) Possible mechanism for photocatalytic oxidation of tetracycline hydrochloride upon MX@MIL-125 [51].

The porous structure of Ti-MOFs provides abundant adsorption sites and transport pathways for pollutants, enabling them to rapidly diffuse to the catalyst surface and contact with the active sites [128]. For instance, novel pleated MX@MIL-125 (Ti) was prepared by synthesizing Ti-MOF on TiC MXene via a hydrothermal method [129]. The presence of O-Ti-O species and graphitic carbon enhanced photosensitivity and catalytic activity. MX@MIL-125 (Ti) presented excellent catalytic performance, in which over 90% of Rhodamine B was eliminated within 60 min and greater than 75.0% of tetracycline hydrochloride was removed within 4 h (Figure 8a,b) [130].

Titanium oxide clusters in Ti-MOFs serve as active sites for the photocatalytic degradation of pollutants. Introducing additional active sites or performing modifications can further enhance photocatalytic performance. For example, Surib et al. [131] prepared a series of bimetallic Ti-MOFs containing silver ions (Ag^+^), iron ions (Fe^3+^), and zinc ions (Zn^2+^) by an ion-exchange strategy and used them in the removal of 2-chlorophenol under natural sunlight irradiation. All the modified Ti-MOFs exhibited higher activity and the Fe^3+^-modified Ti-MOFs achieved the highest conversion rate. The enhancement in activity should be attributed to the new energy levels formed by metal ions between the Ti-MOF bandgaps, which narrowed the bandgap and enhanced light absorption. Similarly, an amine-functionalized Fe/Ti-based bimetallic metal–organic framework (Fe/TiMOF-NH_2_) was developed using a one-pot solvothermal method. This photocatalyst was proved to be particularly effective in the photocatalytic degradation of azo dye Orange II [132].

### 5.4. Photocatalytic Sensors

Ti-MOF photocatalytic sensors take full advantage of the photocatalytic properties of Ti-MOFs to detect various pollutants, organic compounds, and gases through light-induced reactions that generate detectable signals. Consequently, they have gained significant attention due to their high sensitivity, selectivity, and ability to operate under visible light [38,124,133,134].

The signal of Ti-MOF photocatalytic sensors can be improved by the enhanced photocatalytic active sites as a result of the incorporation of metal ions. NU-2300 was a kind of MOF centered on titanium–oxygen clusters, whose open coordination environment enabled precise metal ion doping (e.g., Ni^2+^). Synergistic catalytic centers were therefore formed by the combination of electron-conjugated Ti-O clusters with the doped metals. Photo-generated electrons transferred from the HOMO of organic ligands to the LUMO of the Ti-O cluster and rapidly migrated to the doped metal sites, which effectively suppressed the recombination of electrons and holes [123]. Such a structural advantage results in outstanding performance in a redox-based sensing system. For instance, the synergistic catalytic center efficiently facilitates O_2_ to form oxide active species, which accelerates the pollutant oxidation and degradation in sulphur-containing organic pollutants [135]. Thus, highly sensitive detection can be achieved through changing the electrical signal [136].

**Figure 9:**
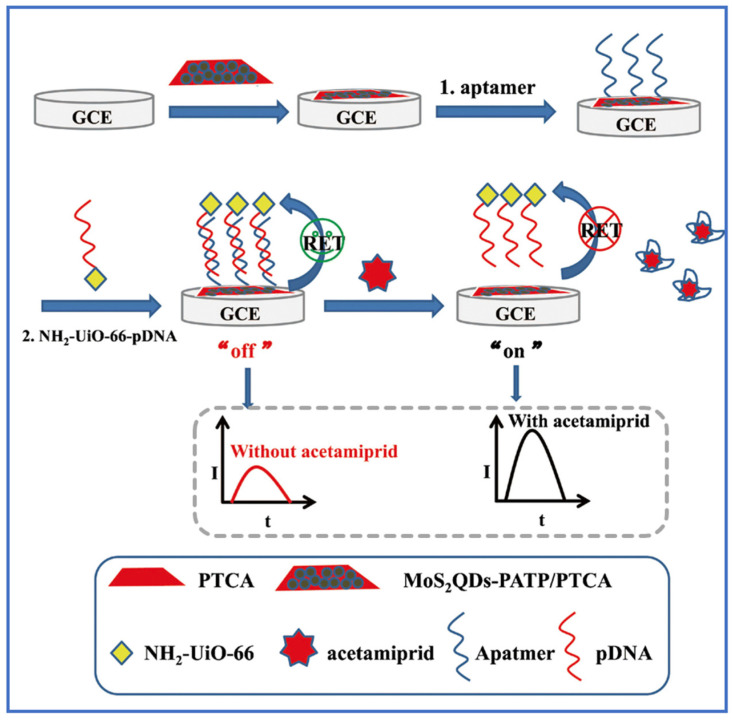
Schematic illustrating the fabrication of the “off–on” ECL acetamiprid aptasensor [136].

A rigid framework is compatible with the composite structures and thus enhances their sensing selectivity and stability. NH_2_-UiO-66 is composed of Zr^4+^ and terephthalic acid ligands and features a rigid cubic structure. Although Zr is the core metal site, Ti-active sites or Ti-doped UiO-66 derivatives can be constructed via post-synthesis modification [130]. The most prominent property of the material is its exceptional chemical stability owing to high crystallinity and the synergistic interaction between surface amino (-NH_2_) functional groups and the porous structure. In practical applications, these structural advantages are fully exploited. For instance, the rigid UiO-66 framework provides stable support for the TiO_2_ coating in a core–shell structure NH_2_-UiO-66@TiO_2_. An efficient electron transfer pathway was constructed within the heterojunction interface. The photo-generated electrons transfer from the conduction band of NH_2_-UiO-66 to the conduction band of TiO_2_, while holes remain in the valence band of NH_2_-UiO-66. Subsequently, they react with O_2_ and H_2_O to generate active species ·O_2_^−^ and ·OH, respectively. This “spatially separated charge utilization” model significantly enhances the oxidation efficiency of VOCs like toluene. Its response value was 1.48 times that of pure NH_2_-UiO-66, with no significant performance degradation after four cycles, highlighting that structural stability is helpful in enhancing sensing practicality [137]. Meanwhile, a novel aptasensor has been fabricated based on the resonance energy transform system from MoS2QDs-PATP/PTCA (donor) to NH_2_-UiO-66 (acceptor). The electrochemiluminescence (ECL) signal of PTCA was greatly amplified due to the decoration of MoS2QDs-PATP, and the NH_2_-UiO-66 was utilized to label the signal probe DNA (pDNA), which hybridizes with the exposed aptamer anchored on the surface of MoS2QDs-PATP/PTCA. With the target acetamiprid, the specific binding of acetamiprid to the aptamer causes the connection between the donor and the acceptor to be interrupted, producing an “on” ECL signal (Figure 9).

## 6. Conclusions and Outlook

Developing high-performance photocatalysts holds significant importance for efficiently harnessing solar energy and removing pollutants against the backdrop of dwindling global fossil energy reserves and worsening environmental pollution. In recent years, significant advances have been achieved in the rational design, controllable synthesis, and photocatalytic application of Ti-MOFs, particularly in the fields of solar fuel production and environmental remediation. Ti-MOFs not only inherit the exceptionally high specific surface area of MOFs but also exhibit great potential in heterogeneous catalysis due to their unique structural flexibility and tunability. Researchers have developed various approaches for the synthesis of Ti-MOFs, including solvothermal synthesis, mechanical milling, microwave-assisted synthesis, supercritical fluid synthesis, and electrochemical methods. Certain techniques can enhance the synthesis efficiency of Ti-MOFs, and others can increase their structural diversity and tunability. Different approaches, like ligand functionalization, ion doping, heterostructure construction, and cocatalyst loading were used to enhance the light absorption capacity, charge transfer efficiency, and stability of Ti-MOFs. A series of Ti-MOFs have shown high efficiency in the photocatalytic degradation of organic pollutants, water splitting for hydrogen production, and carbon dioxide reduction and photocatalytic sensors. This provides important information for the design and preparation of advanced photocatalysts and sheds new light on the application of photocatalysis.

Despite achieving great progress on Ti-MOFs, two challenges, including complicated synthesis and limited stability, have limited their development. The synthesis of Ti-MOFs must resolve difficulties in structural control and harsh reaction conditions because of the requirement of a high specific surface area and flexible frameworks. One-step synthesis attempts may fail under certain conditions, such as improper reaction parameters or reactant control. Additionally, stability issues pose significant challenges in practical applications. Therefore, developing structurally stable titanium-containing multi-level porous MOFs is crucial for their practical application. Challenges also exist in photocatalytic applications. Despite their high specific surface area and flexible structures, aspects such as light utilization efficiency, charge recombination, photocatalytic performance, and specific design for certain reactions still require continuous improvement. To overcome these challenges, in-depth research is required across multiple fronts: selecting appropriate synthesis methods, optimizing reaction conditions, and precisely controlling product structure. This comprehensive approach will improve the quality of Ti-MOFs and expand their practical application.

## Data Availability

No new data were created or analyzed in this study. Data sharing is not applicable to this article.
